# Neurophysiological assessment of central sensory perception among children with bilateral spastic cerebral palsy

**DOI:** 10.6026/973206300220357

**Published:** 2026-01-31

**Authors:** Sangeeta Gupta, Anchala Bhardwaj, Gaurav Gupta

**Affiliations:** 1 Department of Physiology, All India Institute of Medical Sciences (AIIMS) Gorakhpur, Uttar Pradesh, India; 2 Department of Paediatrics, All India Institute of Medical Sciences (AIIMS) Gorakhpur, Uttar Pradesh, India; 3 Department of General Surgery, All India Institute of Medical Sciences (AIIMS), Gorakhpur, Uttar Pradesh, India

**Keywords:** Cerebral palsy, somatosensory evoked potentials, visual evoked potentials and brainstem auditory evoked potentials, spastic, sensory

## Abstract

Somatosensory deficits have gained growing attention in cerebral palsy; therefore, it is of interest to evaluate the central perceptual abnormalities in children with bilateral spastic cerebral palsy (CP). Seventy children (35 children with CP and 35 controls, age-range: 6 months-10 years) were evaluated by somatosensory evoked potentials (SSEP), visual evoked potentials (VEP) and brainstem auditory evoked potentials (BAEP) and the association with electroencephalography (EEG), clinical and radiologic findings was sought for. Tibial SSEPs and median SSEPs revealed abnormal cortical response in 77.14 % (27 of 35) and 65.22 % (15 of 23) respectively, 14.29 % (5 of 35) had abnormal BAEP, 18 of 35 (51.4 %) had abnormal VEP, 14 patients (40 %) demonstrated abnormal EEG and 91.43 % had abnormal brain magnetic resonance imaging (MRI). Abnormal tibial SSEPs were statistically correlated with abnormal EEG (p=0.005) and perinatal asphyxia (p=0.01) while abnormal median SSEPs were statistically correlated with abnormal VEP (p=0.0098) and perinatal asphyxia (p=0.03) (chi-square test). Evidences of sensory cortical involvement in CP can help in designing better treatment plans. Cortical SSEPs may further be evaluated in prospective studies to assess their potential utility as a prognostic tool in children with CP.

## Background:

Cerebral palsy (CP) represents the group of permanent non-progressive motor disorders. Somatosensory system has interestingly been reported to be associated with motor disorders in cerebral palsy [1, 2- 3]. Somatosensory evoked potential (SSEPs) as non-invasive method of investigating central as well as peripheral nervous system can be particularly useful in infants and young children in whom the clinical sensory examination is often difficult and unreliable [4]. In addition, disturbances in central perception of sensory stimulus can be investigated by visual evoked potentials (VEPs) and brainstem auditory evoked potentials (BAEPs) in young children with cerebral palsy in non-invasive manner. Flash VEP has been found to be a useful measure which demonstrated significant association with clinico-radiological parameters [5]. Researchers have also demonstrated deranged SSEPs in children with cerebral palsy [6]. Disruption of sensory tracts, if present might indicate the presence of sensory impairment and also sensory-motor integration deficits in children with cerebral palsy. The radiological investigation can provide the information about the nature and timing of the brain lesion in cerebral palsy [7]. However, the region of present research despite with higher rates of disability in the country and considerable incidence rates of CP, lacks substantial data investigating the same [8, 9- 10]. Most of the data emerge from the developed countries [11, 12]. Teflioudi *et al.* (2011) studied 51 children with bilateral spastic cerebral palsy and reported abnormal somatosensory evoked potentials from median and tibial nerves. The findings could be correlated with abnormal visual evoked potentials and history of perinatal/neonatal infections [13]. Similar findings were demonstrated in SSEPs by posterior tibial nerve stimulation in children with cerebral palsy by Hirayama *et al.* (1999) [6]. Park *et al.* (2002) demonstrated changes in cortical SSEPs after botulinum toxin type A injection in an intervention study in 12 children with spastic cerebral palsy [14]. Vargiami *et al.* (2008) studied 72 children with bilateral spastic cerebral palsy (mean age 3 years) and suggested that flash VEP showed significant correlation with epilepsy and periventricular leukomalacia in MRI [5]. The data from Indian population, however, is scarce. Kothari *et al.* (2010) investigated the relationship between abnormal VEP and BAEP findings with different clinical parameters in children with spastic cerebral palsy [9]. Ansari *et al.* (2016) have obtained auditory brainstem responses for 50 children with spastic CP in the age range 3 to 12 years [10]. The results were subsequently correlated with clinical and neuroradiological findings, additional impairments and disabilities. Therefore, it is of interest to investigate somatosensory and other sensory perceptual integrity in spastic CP in order to find the extent of the brain damage and possible pathophysiologic mechanism contributing to the same.

## Materials and Methods:

Seventy participants (35 children with spastic CP and 35 controls, age-range: 6 months-10 years) were studied for a period of one year. Sample size calculation was done based on the differences in the mean (effect size) from the previous similar study, with power of 80% and 1.96 as the level of statistical significance [13]. The inclusion criteria for the study group were the patients with bilateral spastic cerebral palsy in the age group of 6 months-10 years. The exclusion criteria for the study group were the subjects with otological diseases and peripheral nerve injury. Patients underwent neurophysiological testing and brain MRI. SSEP, VEP and BAEP were performed on Neuro-MEPω EMG and EP digital neurophysiological system software in Neurophysiology laboratory, Department of Physiology, AIIMS, Gorakhpur. Written informed consent from the parents of the children was obtained before the test. Methodology for the test was as recommended by guidelines by American Clinical Neurophysiology Society [15, 16-17]. Children were sedated with oral chloral hydrate (25-75 mg/kg). Preparation of scalp skin was done prior to the electrode application. Standard disc surface electrodes were placed according to the International 10/20 system of electrode placement.

## SSEP:

Median and tibial somatosensory evoked potentials were recorded. A recording in response to unilateral median nerve stimulation was obtained from contralateral cortex CP3 referred to Fz. All the components (N9, 13, 18 and 20) were measured via four channel recording. The cortical component N20 was evaluated. For tibial SSEP, P37 (CPz referred to Fpz) was evaluated. Central conduction time for both median SSEP (N13-N20) and tibial SSEP (N20-P37) was measured.

## VEP:

Flash VEP was recorded with active electrode at Oz, reference electrode at CZ and ground electrode at Fpz. The method of presentation of the stimuli was by means of goggles (monocular stimulation). The signals were amplified and filtered with a system band pass filter of 2-100 Hz. P100 latencies; N75-P100 amplitude, interocular latency difference and interocular amplitude ratio were measured.

## BAEP:

BAEP was recorded with active electrode placed at mastoid (M1 or M2), reference electrode at Cz and ground electrode at Fpz. with Monaural stimulation was done with intensities ranging from 110 to 20 decibel SPL (sound pressure level). Stimulation was delivered by head-phones. BAEP records for absolute latencies of I, III, V waves, interpeak latencies (I-III, III-V, I-V) and ratio of V to I amplitude (V:I) were evaluated.

## EEG:

EEG was conducted with bipolar and referential montages. The responsiveness of the background activity was assessed. EEG response to activation procedures (photic stimulation and hyperventilation) was recorded.

## MRI:

Was recorded in axial T1, T2, FLAIR, sagittal T1, coronal FLAIR and diffusion-weighted imaging sequences.

## Statistical analysis:

Chi2 test, Point biserial correlation, Kendall Tau rank correlation and Pearson correlation were used for the statistical correlation with the qualitative and quantitative variables. Independent sample t-test was employed for comparing group means. p<0.05 was considered as statistically significant.

## Results and Discussion:

The demographic and clinical variables of the children with bilateral spastic CP were measured (Table 1). Quadriplegic type constituted 65.71 % while 34.29 % were diplegic type. Level 5 constituted the majority (40 %) (Gross motor function classification system). Perinatal asphyxia (PNA) was the most common risk factor associated (57.14 %) followed by prematurity (51.43 %) among the patients. The significant contribution of PNA for cerebral palsy in children has well been stated previously [18]. Abnormal IQ (80.65 %) was the most common comorbidity associated, followed by epilepsy (51.43 %), which concords with the previous literature [19, 20- 21]. Mainly the cerebral cortical injury (with injury to the basal nuclei, thalamus and cerebellum playing an additional role) has been implicated in the intellectual disabilities in children with CP [22].

**Figure 1:**
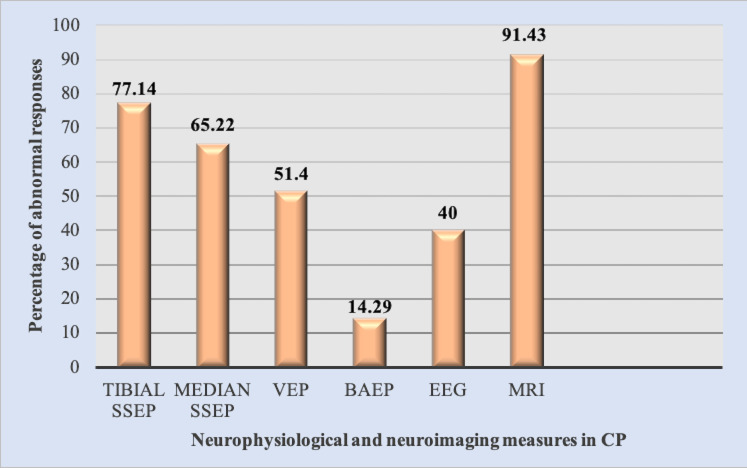
Abnormal neurophysiological and neuroimaging findings in patients with CP. SSEP: somatosensory evoked potentials; VEP: visual evoked potentials; BAEP: brainstem auditory evoked potentials; MRI: magnetic resonance imaging; CP: cerebral palsy

**Figure 2:**
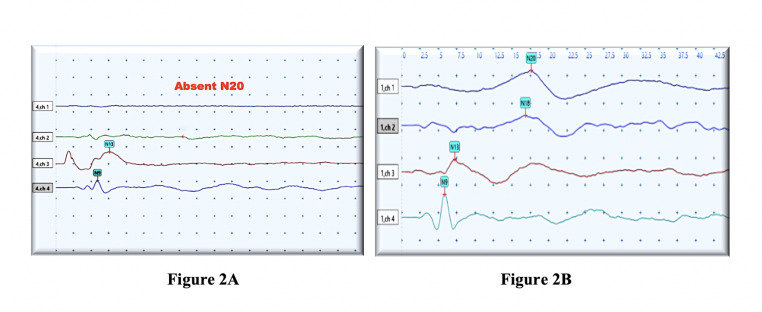
(A)Representative median SSEP record of a 2-year-old male with spastic CP depicting absent cortical response (N20) (four channel recording) (B) Representative median SSEP record of a 2-year-old normal male child depicting normal cortical response (N20) (four channel recording) sensitivity: 5µv, sweep speed: 2.5 ms. µv: microvolt; ms: milliseconds; SSEP; somatosensory evoked potentials

Tibial SSEPs and median SSEPs revealed abnormal cortical response in 77.14 % (27 of 35) and 65.22 % (15 of 23) respectively, 14.29 % (5 of 35) had abnormal BAEP, 18 of 35 (51.4 %) demonstrated abnormal VEP, 14 patients (40 %) had abnormal EEG and 91.43 % had abnormal MRI (Figure 1). Periventricular leukomalacia was the major finding in brain MRI. The above findings are in line with a previous similar study by Teflioudi *et al.* (2011) [13]. Most common abnormality noted in median and tibial SSEP were absent cortical responses (80 %) followed by increase in the central conduction time which conforms to previous studies [13, 23] (Figure 2). VEP findings demonstrated delayed P100 latency. BAEP records showed increased threshold, prolongation of absolute latency of wave V, interpeak latencies of III-V and diminished V-I ratio as abnormal variables as compared to the controls [9]. EEG records had focal epileptiform discharges as the most common abnormal finding [22]. Abnormal tibial SSEPs were statistically correlated with abnormal EEG (p= 0.005) and perinatal asphyxia (p= 0.01) (Table 2) while abnormal median SSEPs were statistically correlated with abnormal VEP (p=0.0098) and perinatal asphyxia (p=0.03) (chi-square test) (Table 3). The results well conform to a previous similar study, however, with regard to the risk factor correlation, they have reported perinatal infections as statistically significantly associated risk factors with median SSEPs [13]. No statistical association between age (Pearson correlation/Point biserial correlation), sex, type of bilateral spastic cerebral palsy, GMFCS level (chi-square test/Kendall Tau rank correlation) and abnormal IQ (Pearson correlation/point biserial correlation) (p>0.05) was found. Prematurity and perinatal infections (chi-square test/point biserial), abnormal MRI and abnormal BAEP findings (chi-square test) were also not found to be correlated with SSEP variables (p>0.05) (Table 2 and Table 3). High proportion of abnormal SSEP documented in the study is consistent with the previous literature [10, 11, 12, 13-14]. Alignment of somatosensory tracts close to the susceptible brain areas may explain the findings [23]. Also, influence of spasticity on the cortical somatosensory evoked potential responses has been interestingly implicated [14]. Correlation of abnormal VEP and SSEP has also been explained on the basis of involvement of parietal lobe and internal capsule fibers in CP [13]. Correlation of abnormal cortical SSEP and EEG strengthens the existence of selected damage in the CNS. Both VEP and EEG have previously been found to be valuable tools to assess the neurological outcome in high-risk neonates. Hence, it seems plausible that evaluating cortical SSEP in children with abnormal EEG and abnormal VEP may be valuable in assessing sensory impairment and also in assessing the neurological outcome in CP in follow-up studies.

## Conclusion:

Data shows impairment of central perception of the sensory stimulus in children with spastic CP. Data on sensory cortical involvement in cerebral palsy can also help in designing better treatment approaches based on sensory integration therapy/occupational therapy. Longitudinal studies evaluating cortical SSEPs in children with spastic CP may further assess the potential utility of cortical SSEP as a prognostic tool in children with CP.

## Advancement to knowledge:

The study strengthens the evidence of central sensory deficits in children with spastic cerebral palsy which has not been extensively investigated yet. The study brings up the plausibility of cortical SSEPs being evaluated further as a non-invasive prognostic tool for investigating the long-term neurological outcome in children with spastic CP.

## Figures and Tables

**Table 1:** Demographic and clinical variables of the study participants (spastic CP) (total n=35)

**S. No.**	**Variable**		**n**	**%**
1	Age	6 months-5 years	18	51.43
		6-10 years	17	48.57
2	Gender	Male	19	54.29
		Female	16	45.71
3	Spastic CP type	Quadriplegic	23	65.71
		Diplegic	12	34.29
4	Functional level of motor skills (GMFCS)	Level I	4	11.42
		Level II	5	14.29
		Level III	5	14.29
		Level IV	7	20
		Level V	14	40
5	Risk factors	Prematurity	18	51.43
		Perinatal asphyxia	20	57.14
		Perinatal infection	7	20
6	Comorbidities	Epilepsy	14	40
		Abnormal IQ	25*	80.65
		Visual dysfunction	18	51.43
		Hearing disorders	5	14.29
		Orthopaedic disorders	14	40
*Wechsler Preschool and Primary Scale of
Intelligence for children (WPPSI) and The Wechsler
Intelligence Scale for Children-fifth edition
(WISC-V) in total 31out of 35 children with CP.
n: number; %: percentage; CP: cerebral palsy;
GMFCS: Gross motor function classification system;
IQ: Intelligence Quotient.

**Table 2:** Statistical association of abnormal tibial SSEPs with other neurophysiological, radiological and clinical variables

**S. No**	**Variable**	**Chi-square value**	**p-value**
1	Abnormal EEG	7.81	0.0052**
2	Abnormal VEP	3.5	0.06
3	Abnormal BAEP	1.75	0.19
4	Abnormal MRI	0.03	0.86
5	Perinatal asphyxia	6.6	0.01*
6	Prematurity	3.54	0.059
7	Perinatal infection	1.37	0.24
*p<0.05, **p<0.01 -
SSEP: somatosensory evoked potentials;
EEG: electroencephalography;
VEP: visual evoked potential;
BAEP: brainstem-auditory evoked potentials;
MRI: magnetic resonance imaging.

**Table 3:** Statistical association of abnormal median SSEPs with other neurophysiological, radiological and clinical variables

**S. No**	**Variable**	**Chi-square value**	**p-value**
1	Abnormal EEG	3.55	0.06
2	Abnormal VEP	6.67	0.0098**
3	Abnormal BAEP	0.99	0.32
4	Abnormal MRI	0.04	0.84
5	Perinatal asphyxia	4.66	0.03*
6	Prematurity	1.34	0.25
7	Perinatal infection	0.72	0.4
*p<0.05, **p<0.01 -
SSEP: somatosensory evoked potentials;
EEG: electroencephalography;
VEP: visual evoked potential;
BAEP: brainstem-auditory evoked potentials;
MRI: magnetic resonance imaging.
